# Defective Induction of COX-2 Expression by Psoriatic Fibroblasts Promotes Pro-inflammatory Activation of Macrophages

**DOI:** 10.3389/fimmu.2019.00536

**Published:** 2019-03-20

**Authors:** Jorge Arasa, María Carmen Terencio, Rosa María Andrés, Asunción Marín-Castejón, Francisca Valcuende-Cavero, Miguel Payá, María Carmen Montesinos

**Affiliations:** ^1^Instituto Interuniversitario de Investigación de Reconocimiento Molecular y Desarrollo Tecnológico (IDM), Universitat Politècnica de València, Universitat de València, Valencia, Spain; ^2^Departament of Pharmacology, Faculty of Pharmacy, Universitat de València, Valencia, Spain; ^3^Department of Dermatology, University Hospital La Plana, Vila-real, Spain; ^4^Predepartamental Unit of Medicine, Universitat Jaume I, Castellón, Spain

**Keywords:** cyclooxygenase, fibroblasts, psoriasis, macrophages, inflammation

## Abstract

Fibroblasts play an important role as members of the innate immune system through the secretion of COX-2-derived inflammatory mediators such as prostaglandin E_2_ (PGE_2_). However, it has been described that dermal fibroblasts behave like mesenchymal stem cells reducing lymphocyte recruitment and dendritic cell activation through PGE_2_ release. As the role of fibroblasts in psoriasis remains poorly characterized, in the present study we have evaluated the possible influence of PGE_2_ derived from dermal fibroblasts as modulator of the immune response in psoriatic skin. Our results indicate that under inflammatory conditions, psoriatic fibroblasts showed defective induction of COX-2, which resulted in diminished production of PGE_2_, in contrast to healthy fibroblasts. This phenotype correlated with deficient c-Jun N-terminal kinase (JNK) activation, in accordance with the hypothesis that alterations in members of the JNK pathway are associated with psoriasis. Furthermore, conditioned medium from psoriatic fibroblasts promoted the polarization of monocytic cells toward a pro-inflammatory profile, effect that was mimicked in healthy fibroblasts after pre-incubation with indomethacin. These results are consistent with a prominent role of dermal fibroblasts in the regulation of inflammatory response through the participation of COX-derived metabolites. This resolutive behavior seems to be defective in psoriatic fibroblasts, offering a possible explanation for the chronification of the disease and for the exacerbation triggered by nonsteroidal anti-inflammatory drugs (NSAIDS) such as indomethacin.

## Introduction

Psoriasis is a chronic skin inflammatory disease characterized by the appearance of scaly plaques as result of the interplay of genetic, environmental, and immunological factors (1, 2). From a pathophysiological perspective, psoriasis is largely caused by an imbalance between the local immune response and its regulatory mechanisms, mainly involving lymphocytes, dendritic cells, and keratinocytes (3). Although the participation of other resident cells such as Langerhans cells or macrophages has also been recognized (3, 4); the role of fibroblasts, the main cell type in the dermis, remains relatively poorly characterized.

Fibroblasts are stromal cells, responsible for the synthesis and remodeling of extracellular matrix components, which regulate homeostasis and play a critical role during tissue development, differentiation, and repair (5). They are also considered members of the innate immune system and could contribute to the pathogenesis of several diseases, such as rheumatoid arthritis or tumor development, through the secretion of cytokines, chemokines, or eicosanoids (6–8). Interestingly, depending on their location, fibroblasts are able to display either pro-inflammatory or anti-inflammatory properties and influence leukocyte recruitment. Thus, synovial fibroblasts develop a direct inflammatory phenotype in the inflamed synovium, whereas dermal fibroblasts are more functionally similar to mesenchymal stem cells, limiting cytokine sensitivity of vascular endothelium, lymphocyte recruitment and dendritic cell activation (5, 9, 10). These regulatory effects are in part mediated by classically considered pro-inflammatory mediators such as prostaglandin E_2_ (PGE_2_), which is highly produced by healthy dermal fibroblasts (11, 12).

PGE_2_, generated by the action of cyclooxygenases (constitutively active COX-1 and inducible COX-2) on the membrane phospholipid arachidonic acid, is generally recognized as a mediator of active inflammation at early stages. In healthy human fibroblasts, COX-2 is upregulated upon inflammatory stimulation (13), and released PGE_2_ supports the migration of dendritic cells and promotes IL-23 secretion (14, 15). Paradoxically, PGE_2_ can suppress both innate and antigen-specific stimulated immunity in chronic processes by promoting the induction of suppressive IL-10 and reducing the production of pro-inflammatory cytokines such as tumor necrosis factor α (TNF-α) in macrophages (16). Therefore, the role of PGE_2_ in psoriasis remains still controversial. Moreover, administration of non-steroidal anti-inflammatory drugs (NSAIDs) can exacerbate the symptoms of the disease (17, 18).

There is evidence that fibroblasts isolated from diseased tissues exhibit phenotypic differences compared with fibroblasts taken from normal tissues (19). Accordingly, we recently described that dermal fibroblasts obtained from psoriatic plaques display a defective activation of the c-Jun N-terminal kinase (JNK) pathway that leads to diminished cytokine production (20). In the present study, we sought to further investigate the role of fibroblasts as modulators of the immune response in the skin and put in the spotlight the possible participation of altered psoriatic dermal fibroblasts in the chronification and exacerbation of this disorder. To do so, we determined if fibroblast-released factors influence the polarization of macrophages.

## Materials and Methods

### Materials

Interleukin-1β (IL-1β) was obtained from Peprotech (Rocky Hill, NJ). Polyclonal rabbit antibody against COX-2 was from Millipore (Temecula, CA). Polyclonal goat anti-rabbit Immunoglobulins/HRP were from Dako (Glostrup, Denmark). Diaminobenzidine (DAB) was from Vector (Burlingame, CA). Monoclonal antibodies against phospo-NFκB p65 (Ser536), phospho-p38 (Thr180/Tyr182), phospho-ERK1/2 (Thr202/Tyr204), phospho-SAPK/JNK (Thr183/Tyr185), and RelB were from Cell Signaling Technology (Beverly, MA). 12-*O*-tetradecanoyl phorbol 13-acetate (TPA), 3-(4,5-dimethylthiazol-2-yl)-2,5-diphenyltetrazolium bromide (MTT) and other reagents were from Sigma-Aldrich (St. Louis, MO).

### Isolation and Culture of Primary Human Fibroblasts

All protocols and procedures were approved by the University of Valencia Ethical Committee (number of procedure: H1396456196160) and carried out according to the Declaration of Helsinki Principles. Tissue samples were obtained from programmed clinical interventions and donors signed the informed consent to use the samples for research purposes.

Primary fibroblasts were obtained from freshly resected foreskins of 14 adult healthy donors (25.8 ± 2.3 years, Caucasian) and from lesional skin biopsies taken for diagnosis purposes of 14 psoriatic patients (42.9 ± 15.7 years, Caucasian, 10 men and 4 women, PASI = 10.2 ± 15.9) (Table S1). Patients had received no topical treatment for at least 2 weeks and no systemic treatment for at least 4 weeks before the procedure and diagnosis of plaque-type psoriasis was confirmed by histological analysis. Skin samples were cut into small pieces and incubated overnight at 4°C in 0.5% dispase II. Epidermis was removed and dermis was digested during 90 min at 37°C in 0.1% collagenase IA. Suspension was passed through a 70 μm filter and centrifuged. Cells obtained directly after digestion were cultured at 37°C, 5% CO_2_ in DMEM/F12 HAM medium supplemented with 10% FBS (Biowest, Nuaillé, France) and 1% penicillin/streptomycin. After reaching 80–90% confluence, cells were passaged using 0.25% trypsin. Cultured cells from freshly isolates were used between passages 2 and 5.

### MTT Assay and Radioimmunoassay

Cells were seeded in 24-well culture plate (50,000 cells/well). Next day, medium was replaced and cells were stimulated with 2.5 ng/ml IL-1β or 1 μg/ml TPA. After 24 h, supernatants were collected and remaining fibroblasts were used to determine the reduction of 3-(4,5-dimethylthiazol-2-yl)-2,5-diphenyltetrazolium bromide (MTT) to formazan. PGE_2_ release was determined in the supernatants by radioimmunoassay (RIA) (21).

### Western Blotting

To assess the induction of COX-2, cells were seeded in 6 cm Petri dishes (500,000 cells/dish). Next day, medium was replaced and cells were stimulated with 2.5 ng/mL IL-1β or 1 μg/mL TPA during 24 h. IL-1β treated supernatants were collected (conditioned medium) and cells whole protein extraction was carried out with RIPA buffer (50 mM Tris, 150 mM NaCl, 0.5% Sodium Deoxycholate, 1% Triton X-100) containing an antiprotease cocktail (21). RelB expression was measured at 6 h post stimulation.

To study the phosphorylation of MAPKs and NF-κB, cells were seeded in 6 cm Petri dishes (500,000 cells/dish) until confluence and subsequently starved during 6 h with medium without FBS. After 15-min stimulation with 2.5 ng/ml IL-1β or 1 μg/ml TPA, whole cell protein extraction was carried out with RIPA buffer containing an antiprotease cocktail.

Western blot of all lysates was performed as previously described (22). Images were captured with the AutoChemi image analyzer (UVP Inc., Upland, CA). GAPDH was used as a protein loading control.

### Immunocytochemistry

Cells were seeded in 8-wells chambers (50,000 cells/well). Next day, medium was replaced and cells were stimulated during 24 h as above. After 15 min fixation with paraformaldehyde 4% and peroxidase blocking (Dako, Copenhagen, Denmark), cells were incubated 2 h with COX-2 antibody and 1 h with secondary HRP anti-rabbit antibody. Development of the peroxidase staining was performed with DAB and visualized in Leica DM IL LED microscope, using Leica Application Suite (Solms, Germany).

### RNA Extraction and Real-Time Quantitative RT-PCR

Cells were seeded in 6 cm Petri dishes (500,000 cells/dish). When confluence was reached, medium was renewed and cells were stimulated with 2.5 ng/ml IL-1β or 1 μg/ml TPA. Total RNA was extracted using TriPure Isolation Reagent (Roche, Mannheim, Germany). cDNA of COX-2 and GAPDH were obtained using ImProm-II Reverse Transcription System (Promega Corporation, Madison, WI). cDNA of miR-146a and U6 were obtained using TaqMan MicroRNA Reverse Transcription Kit (Applied Biosystems, Foster City, CA).

All cDNAs were quantified via real-time PCR using Taqman 20x Assays-On-Demand (FAM-labeled MGB-probes) gene expression assay mix (Applied Biosystems; assay ID: Hs00153133_m1, Hs00377726_m1, HS99999905_m1, 4427975-000468, and 4395470-001973, respectively) and Taqman Universal PCR Master Mix No AmpErase UNG (Applied Biosystems).

The expression levels were analyzed on a StepOnePlus machine (Applied Biosystems) and normalized to the housekeeping GAPDH or U6.

### Macrophages-Derived THP-1 Culture

THP-1 cells were cultured at 37°C, 5% CO_2_ in RPMI medium supplemented with 10% SBF and 1% penicillin/streptomycin. Differentiation of THP-1 to macrophages was performed seeding THP-1 in 24-well plates (400,000 cells/well) and incubating them with TPA 10 nM during 3 days. Then, adherent cells were washed with DMEM and incubated for 24 h with conditioned media from psoriatic fibroblast (PFCM) or healthy fibroblasts (HFCM) previously stimulated with IL-1β (2.5 ng/ml) during 24 h. In other set of experiments, THP-1 cells were incubated during 24 h with PGE_2_ (30 ng/ml), indomethacin (10 μM), or their combination in the presence or absence of IL-1β (2.5 ng/ml) in DMEM/F12 HAM medium.

### Statistical Analyses

Results are presented as mean ± SD. Statistical analyses were performed using either two-way ANOVA followed by Sidak's multiple comparison test or one-way ANOVA followed by Tukey's multiple comparison test, carried out by GraphPad Prism 4 software (GraphPad Software, Inc., San Diego, CA). A value of *p* < 0.05 was considered statistically significant.

## Results

### Failure to Induce COX-2 Expression Resulted in Reduced Production of PGE_2_ by Dermal Fibroblasts From Psoriatic Plaques

**Figure 1:**
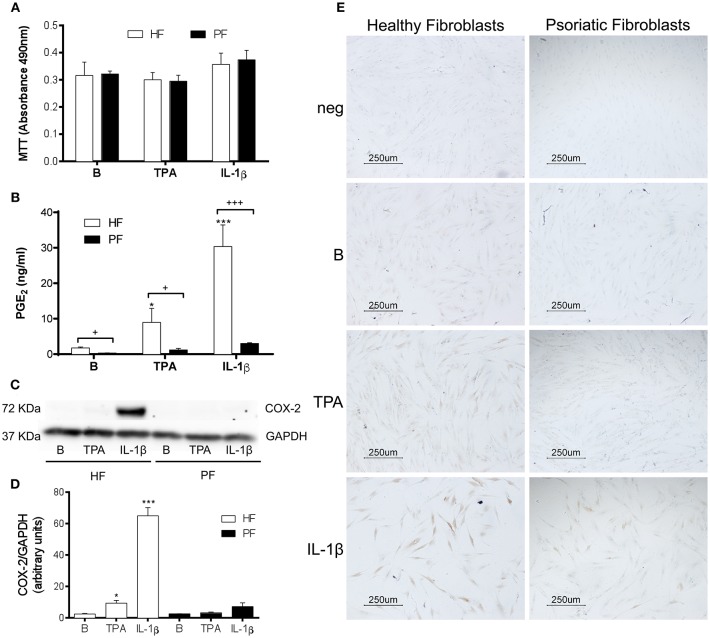
PGE_2_ production and COX-2 expression are decreased in stimulated psoriatic fibroblasts. Cells were treated with 2.5 ng/ml IL-1β or 1 μg/ml TPA for 24 h. **(A)** 3-(4,5-dimethylthiazol-2-yl)-2,5-diphenyltetrazolium bromide (MTT) assay for cell viability (*n* = 4 biopsies) and **(B)** Prostaglandin E_2_ (PGE_2_) determined by radioimmunoassay (*n* = 6 biopsies) were performed in duplicate. **(C,D)** COX-2 protein expression assessed by Western blotting (*n* = 3 biopsies). Data represent mean ± SD. *p < 0.05, ***p < 0.001 vs. unstimulated fibroblasts (B) and +*p* < 0.05, +++*p* < 0.001 vs. healthy fibroblasts (HF) using Sidak's multiple comparison test. PF, psoriatic fibroblasts. **(E)** COX-2 protein expression determined by immunocytochemistry (representative photomicrographs of three independent experiments).

Several studies using fibroblasts obtained from surgical resections of healthy skin suggest that PGE_2_ may contribute to psoriasis pathogenesis by promoting recruitment and activation of T-cells, dendritic cells and monocytes (14, 15). Nonetheless, PGE_2_ also has anti-inflammatory effects that are both potent and context dependent (23). To explore the production of this prostaglandin by plaque-type psoriatic fibroblasts, we selected two different stimuli: IL-1β (2.5 ng/ml), which potently induces COX-2 expression in healthy fibroblasts (24); and the direct protein kinase C (PKC) activator, 12-O-tetradecanoylphorbol-13-acetate (TPA, 1 μg/ml), which triggers epidermal hyperplasia *in vivo* (22) and induces COX-2 expression by a receptor-independent mechanism (25). After discarding the possible cytotoxicity by the MTT assay (Figure 1A), release of PGE_2_ was determined in cell supernatants by radioimmunoassay. Results showed that psoriatic fibroblasts failed to produce a significant increase of PGE_2_ after 24 h stimulation with either stimulus, in contrast to fibroblasts from surgical resections of healthy donors (Figure 1B). It is interesting to note that basal levels of this eicosanoid were also significantly lower in psoriatic than in healthy fibroblasts.

Western blot analysis, performed using the above experimental conditions, confirmed that the lower production of PGE_2_ by psoriatic fibroblasts correlated with a failure to induce COX-2 expression. As seen in Figure 1C, IL1-β markedly induced COX-2 expression in healthy fibroblasts, whereas a non-significant increase was observed in psoriatic fibroblasts (Figures 1C,D). Similar effects were obtained by immunocytochemistry, which only revealed a slight positive response in IL1-β-treated psoriatic fibroblasts compared to the pronounced expression obtained in healthy fibroblast (Figure 1E).

**Figure 2:**
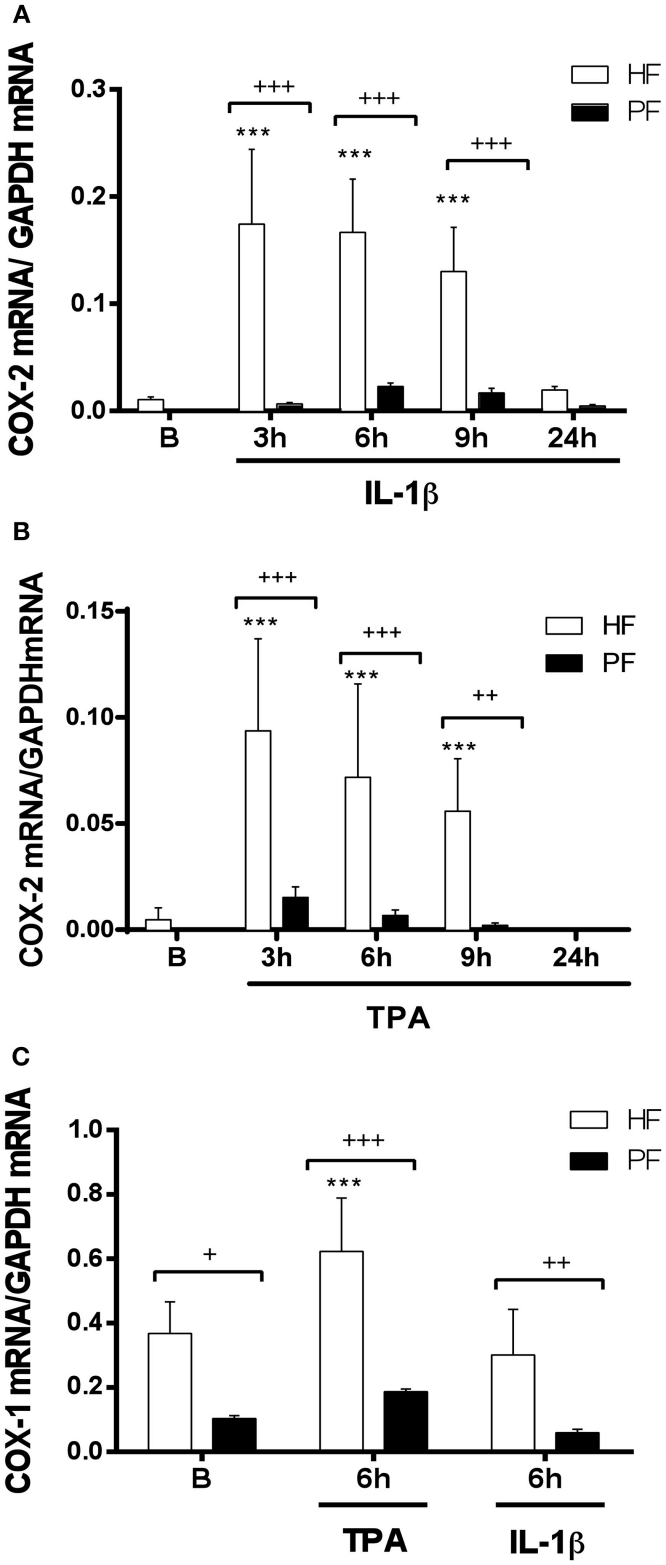
COX-1 and COX-2 mRNA expression is decreased in psoriatic fibroblasts. Cells were treated with 2.5 ng/ml IL-1β **(A)** or 1 μg/ml TPA **(B)** for 3, 6, 9, or 24 h and COX-2 mRNA levels were evaluated by quantitative real-time PCR. **(C)** COX-1 mRNA levels were evaluated by quantitative PCR after IL-1β or TPA stimulation during 6 h. Data represent mean ± SD (*n* = 6 biopsies) of mRNA expression normalized to the housekeeping gene GAPDH and expressed as 2^−ΔΔCT^ values. ***p < 0.001 *vs*. unstimulated healthy fibroblasts (B) and +*p* < 0.05, ++*p* < 0.01, +++*p* < 0.001 *vs*. healthy fibroblasts (HF) using Sidak's multiple comparison test. PF, psoriatic fibroblasts.

To assess the possible deficiency at mRNA expression level, psoriatic and healthy fibroblasts were treated with TPA or IL1-β for 3, 6, 9, and 24 h and COX-2 mRNA was determined by real-time reverse transcriptase PCR (RT-PCR). The slight increase of COX-2 mRNA expression in psoriatic fibroblasts induced by either stimulus at several times was not statistically significant, in contrast to mRNA increase obtained using healthy fibroblasts (Figures 2A,B). Since basal PGE_2_ levels by unstimulated psoriatic fibroblasts were reduced, we determined mRNA expression of the constitutive COX-1. Our results show that TPA was able to induce a significant increase of mRNA levels in healthy fibroblasts, whereas mRNA expression remained similar before and after IL1-β stimulation. Psoriatic fibroblasts followed a similar pattern of expression, but COX-1 mRNA levels were always lower than in healthy fibroblasts (Figure 2C).

### Defective Activation of the JNK Pathway in Psoriatic Fibroblast Would Lead to Reduced COX-2 Expression

**Figure 3:**
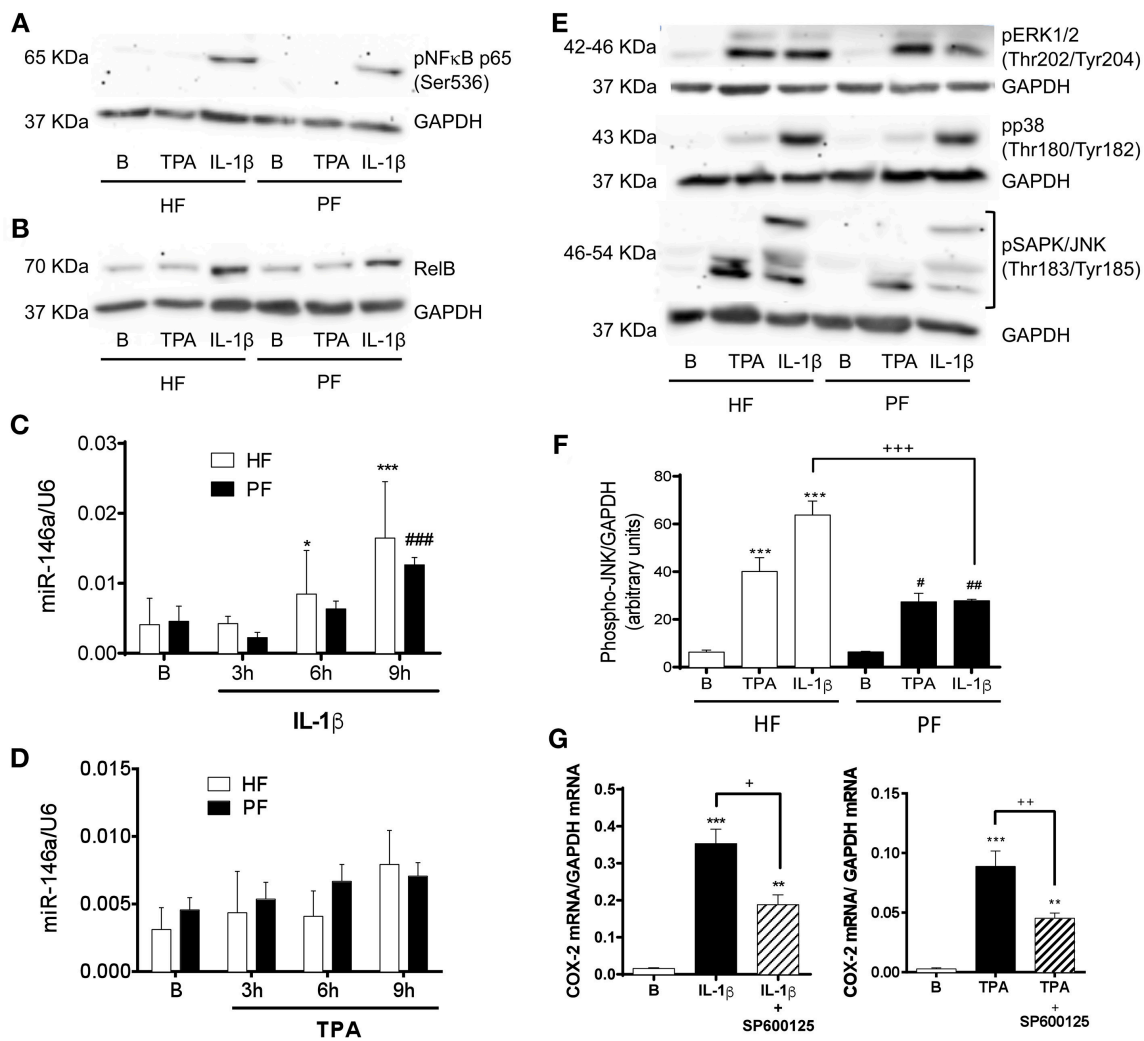
Psoriatic fibroblasts show an altered activation of the SAPK/JNK pathway involved in COX-2 induction. **(A)** Representative immunoblotting image of phosphorylated NF-κB p65 subunit after 15 min treatment with 2.5 ng/ml IL-1β or 1 μg/ml TPA. **(B)** Representative immunoblotting image of phosphorylated NF-κB RelB subunit after 6 h treatment with IL-1β or TPA. **(C,D)** miR146a expression determined by RT-qPCR at 0, 3, 6, or 9 h post stimulation with IL-1β or TPA. Data represent mean ± SD (*n* = 4 biopsies) of 2^−ΔΔCT^ values normalized to the small nucleolar RNA U6. *p < 0.05, ***p < 0.001 vs. non stimulated (B) healthy fibroblasts (HF) and ###*p* < 0.001 *vs*. non stimulated (B) psoriatic fibroblasts (PF) using Sidak's multiple comparison test. **(E)** Representative immunoblotting image of phosphorylated p38, ERK1/2, and SAPK/JNK after 15 min stimulation with IL-1β or TPA. **(F)** Densitometry analysis of phospho SAPK/JNK immunoblots. Data represent mean ± SD (*n* = 4). ***p < 0.001 *vs*. non stimulated (B) healthy fibroblasts (HF); #*p* < 0.05, ##*p* < 0.01 *vs*. non stimulated (B) psoriatic fibroblasts (PF) and +++ *p* < 0.001 vs. healthy fibroblasts (HF) using Sidak's multiple comparison test. **(G)** RT-qPCR for COX-2 mRNA in HF after preincubation with the JNK inhibitor SP600125 (50 μM) during 45 min and the subsequently stimulation with IL-1β (*n* = 6 biopsies) or TPA (*n* = 8 biopsies) during 2 h. Data represent mean ± SD. **p < 0.01, ***p < 0.001 *vs*. unstimulated cells (B); +*p* < 0.05, ++*p* < 0.01 *vs*. IL-1β/TPA stimulated fibroblasts using Tukey's multiple comparison test.

A similar deficit in COX-2 induction and subsequent PGE_2_ production has been described in fibroblasts from other pathological conditions such as asthma (26, 27) and idiopathic pulmonary fibrosis (28, 29). It has been described that COX-2 transcription in nasal fibroblasts is critically governed by mitogen activated protein kinases (MAPKs) and the NF-κB complex (26). However, the non-canonical NF-κB member RelB exerts anti-inflammatory effects in IL-1β-stimulated lung fibroblasts by upregulating miR-146a, which would efficiently degrade COX-2 mRNA (30). Therefore, we sought to determine the canonical and non-canonical NF-κB pathways in psoriatic fibroblasts. Immunoblotting of the phosphorylation of NF-κB p65 after 15 min stimulation with IL-1β or TPA showed the same grade of activation in both, healthy and psoriatic fibroblasts (Figure 3A). We observed as well that RelB was similarly expressed in healthy and psoriatic fibroblasts at basal conditions or after 6 h stimulation (Figure 3B). In addition, miR-146a expression determined by RT-qPCR was induced in a time-dependent manner by IL1-β and TPA, but no differences were found between psoriatic and healthy fibroblasts (Figures 3C,D).

With respect to the possible alteration of MAPKs activation pathways, there were no differences between psoriatic and healthy fibroblasts in the phosphorylation of MAPKs p38 and extracellular signal-regulate kinase (ERK) 1/2. However, both stimuli, IL-1β and TPA, caused less activation of SAPK/JNK in psoriatic fibroblasts than in healthy fibroblasts (Figures 3E,F).

To prove the regulatory role of SAPK/JNK in COX-2 expression by dermal fibroblasts, we preincubated healthy fibroblasts with the JNK inhibitor SP600125 (50 μM) prior to 2 h stimulation with IL-1β or TPA. RT-qPCR analysis showed that SP600125 significantly diminished COX-2 mRNA levels after both stimuli (Figure 3G). These results confirm that SAPK/JNK is directly involved in the COX-2 expression in dermal fibroblasts and suggest that its downregulation could be responsible, at least in part, of the failure in COX-2 induction observed in psoriatic fibroblasts.

### Defective Induction of COX-2 Expression by Psoriatic Fibroblasts Promotes the Polarization of Macrophages to a Pro-inflammatory Phenotype

**Figure 4:**
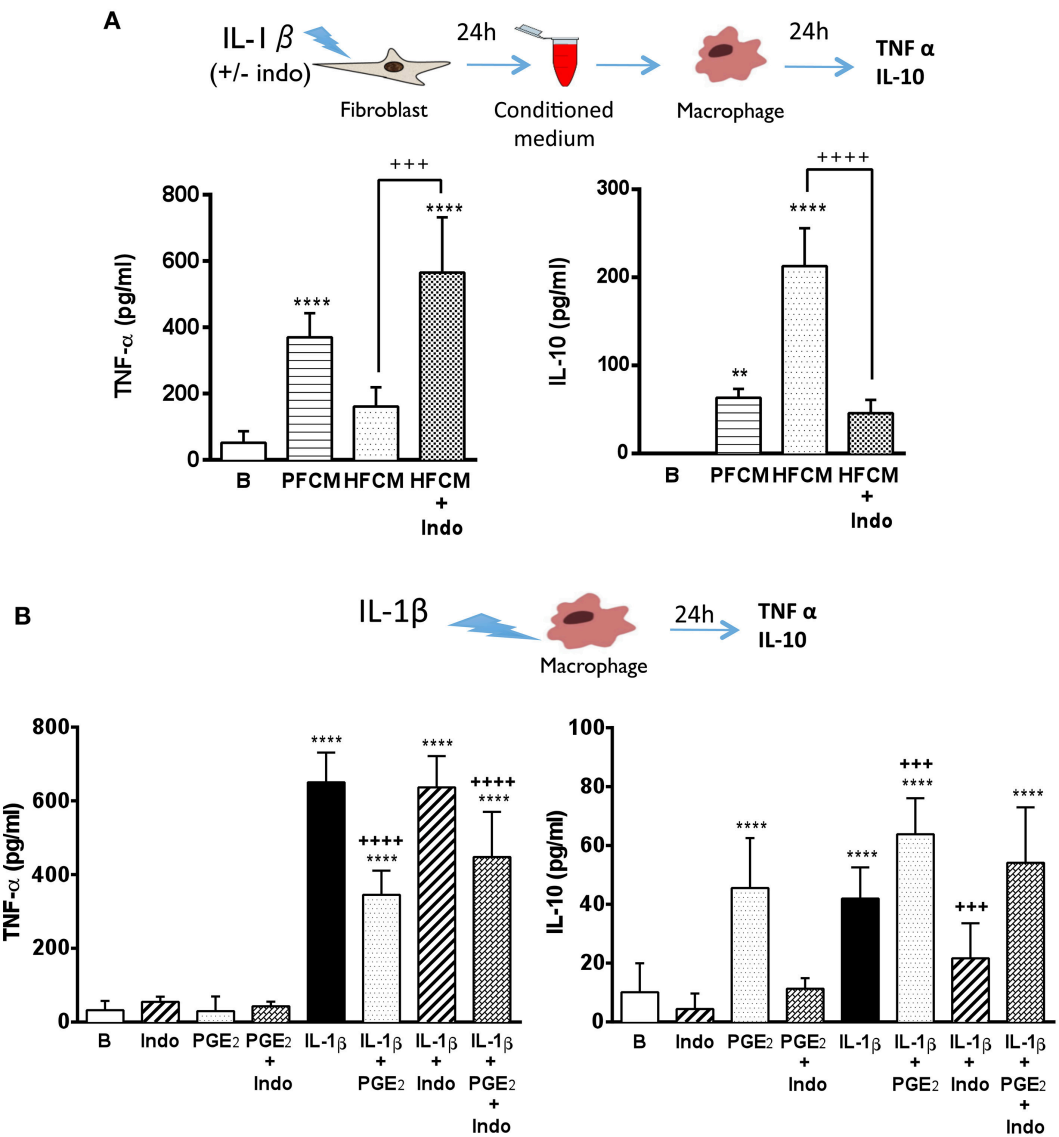
Psoriatic fibroblasts support pro-inflammatory activation of macrophages. **(A)** TNF-α and IL-10 levels in THP-1 derived macrophages incubated during 24 h with conditioned media from IL-1β-stimulated psoriatic fibroblast (PFCM) (*n* = 4 biopsies) or IL-1β-stimulated healthy fibroblasts (HFCM) (*n* = 4 biopsies). When indicated, healthy fibroblasts where pretreated with 10 μM indomethacin (Indo) before stimulation. Data represent mean ± SD. **p < 0.01, ****p < 0.0001 respect untreated macrophages (B); +++*p* < 0.001 ++++*p* < 0.0001 *vs*. macrophages treated with HFCM using Tukey's multiple comparison test. **(B)** TNF-α and IL-10 levels in THP-1 derived macrophages treated during 24 h with PGE_2_ (30 ng/ml), indomethacin (10 μM) or their combination in the presence or the absence of IL-1β (2.5 ng/ml). Data represent mean ± SD (*n* = 6). ***p < 0.001, ****p < 0.0001 *vs*. untreated cells (B). +++*p* < 0.001 respect IL-1β-treated cells using Tukey's multiple comparison test. All conditions were assayed in duplicate.

The interplay between fibroblasts and macrophages is crucial to maintain and to re-establish the integrity of the dermis. Both cell types are key players during the local inflammatory response triggered by tissue injury and also in the subsequent resolution phase (31, 32). Macrophages display different phenotypes depending on the inflammatory environment, leading to the resolution or the aggravation of the inflammatory process (33). In psoriatic lesions, classically activated macrophages represent an important source of TNF-α, a cytokine highly involved in the pathogenesis of the disease (4, 34, 35). Furthermore, a reduction of IL-10 levels has been described in psoriatic lesions (36). Thus, we decided to determine the possible influence of fibroblasts on the macrophage response. For this purpose, conditioned media collected from healthy and psoriatic fibroblasts stimulated during 24 h with IL-1β were added to THP-1-derived macrophage cultures, and the release of TNF-α and IL-10 was determined. Macrophages incubated during 24 h with stimulated psoriatic fibroblast conditioned media (PFCM) exhibited a clear pro-inflammatory profile, producing high levels of TNF-α and low levels of the anti-inflammatory cytokine IL-10 (Figure 4A). In contrast, macrophages incubated with stimulated healthy fibroblast conditioned media (HFCM), produced low levels of TNF-α and high amount of IL-10, suggesting a physiological regulatory profile.

In order to emulate the decreased expression of COX-2 found in psoriatic fibroblasts, the non-selective COX-1 and COX-2 inhibitor, indomethacin (10 μM), was used to treat healthy fibroblasts prior to IL-1β-stimulation, and macrophages were incubated with conditioned medium. Under these conditions, macrophages produced high levels of TNF-α and low levels of IL-10 in a similar manner to macrophages incubated with PFCM (Figure 4A). These results suggested a possible regulatory role of PGE_2_ produced by healthy fibroblasts on macrophage activation, which could be lost in psoriatic fibroblasts due to the decrease in their ability to produce this eicosanoid.

To further demonstrate the effect of PGE_2_, macrophages were directly stimulated with IL-1β in the presence or absence of 30 ng/ml exogenous PGE_2_ (concentration detected in supernatants of healthy fibroblasts after 24 h stimulation with IL-1β, Figure 1B). As shown in Figure 4B, exogenous PGE_2_ induced a regulatory phenotype in stimulated macrophages, reducing the production of TNF-α and prompting the production of IL-10, regardless the presence of indomethacin. Interestingly, pre-treatment with indomethacin supported an inflammatory behavior in stimulated macrophages, maintaining high levels of TNF-α, but blunting the production of IL-10.

## Discussion

Recent studies suggest that tissue-resident stromal cells such as fibroblasts are critically positioned at the cellular and molecular basis for disease persistence, as regulators of the switch from acute resolving to chronic persistent inflammation (5). Fibroblasts from healthy tissues predominantly exhibit a regulatory and immunomodulatory phenotype, whereas fibroblasts from diseased tissues display pro-inflammatory characteristics and promote leucocyte recruitment (10, 12). In the synovial joint, fibroblast-like synoviocytes amplify tissue damage through the production of eicosanoids, cytokines and proteases (37). In certain types of cancers, including colon, breast and prostate, a specific population of cancer-associated fibroblasts (CAFs) strongly induce COX-2 and secrete PGE_2_, which results essential in tumor survival and metastasis (7, 8, 38–40). In psoriatic dermis, a positive COX-2 immunostaining was previously described, although the main cell type involved (inflammatory or stromal cells) remains unclear (15, 41). Thus, we decided to investigate the role of PGE_2_ derived from dermal fibroblast in the pathogenesis of psoriasis. Unexpectedly, our results demonstrated a disability of psoriatic fibroblasts to induce COX-2 and generate PGE_2_ under inflammatory conditions. Even constitutive COX-1 was less expressed in psoriatic than in healthy fibroblasts, in accordance with Niu et al. (42), who described a significant decrease in COX-1 expression by mesenchymal stem cells derived from dermis of psoriatic lesions compared to healthy controls. Interestingly, a similar behavior has been described in other diseases such as idiopathic pulmonary fibrosis and chronic asthma, where the inability of lung fibroblasts to upregulate PGE_2_ in response to a cytokine stimulus seems to contribute to the evolution of airway fibrosis (27, 28, 43).

In view of our results, PGE_2_ derived from healthy fibroblasts would exert a regulatory function, as previously described (16, 23), which is lost in psoriatic fibroblasts, contributing to the lack of control of cellular infiltration and inflammatory response. This hypothesis gets reinforced by the fact that COX-derived mediators released by dermal fibroblasts promote alternative macrophage activation improving wound healing (32). In particular, PGE_2_ has a profound effect on macrophages (44), favoring the conversion of pro-inflammatory macrophages to a more anti-inflammatory phenotype (45). Nevertheless, dermal fibroblasts produce other prostanoids besides PGE_2_, such as PGE_1_, PGD_2_, PGF_2α_, or PGJ_2_ (46, 47), and it has been shown that both PGE_2_ and PGD_2_ from conditioned medium of stimulated myofibroblasts promote an alternative macrophage activation, which is abrogated by indomethacin and selective COX-2 inhibitors, suggesting a principal role of this isoenzyme in wound healing (48).

Our results suggest that COX-derived products of dermal fibroblasts favor the alternative differentiation of macrophages toward a resolutive phenotype, which is defective in psoriatic skin. In support of this hypothesis, a clinical exacerbation of psoriasis after administration of NSAIDs, such as indomethacin and phenylbutazone, has been described; although the use of NSAIDs is rarely discouraged by dermatologists in patients with psoriasis (17). Moreover, in a small clinical trial involving patients with plaque-type psoriasis, topical application of a PGE_2_ gel (5 mg/g, under occlusive dressing) improved certain signs of psoriatic lesions in comparison to gel alone, without completely clearing them (49). The search for the molecular mechanism responsible for the decrease of COX-2 and PGE_2_ in psoriatic fibroblasts led us to detect a failure in the phosphorylation of the SAPK/JNK pathway. In this sense, mutations in different members of the JNK pathway have been associated with psoriasis. Thus, JunB gene is located in the psoriasis susceptibility region PSORS6, and epidermal deletions of JunB and c-Jun in mice lead to psoriasis-like lesions (50). Additionally, it has been described that knock out fibroblasts in JNK compromise the normal differentiation and proliferation of keratinocytes, suggesting a pivotal role of fibroblast-derived soluble factors in the efficiency of wound healing (51). In this context, we recently described that defective production of IL-6 in psoriatic fibroblasts was also related to the downregulation of SAPK/JNK pathway (20). In our studies, we observed similar low levels of phosphorylated MAPKs in unstimulated serum-deprived normal and psoriatic fibroblasts, as previously described by Dimon-Gadal et al. (52). In contrast, Becatti et al. (53) reported an altered phosphorylation of JNK as well as p38 and ERK in basal psoriatic fibroblasts respect to healthy cells, possibly due to different experimental conditions. Interestingly, they reported that psoriatic fibroblasts showed reduced expression and activity of Sirtuin 1 (SIRT1), which was responsible for the loss of protective mechanisms against oxidative stress and redox balance (53). In a similar manner, Guban et al. (54) have also recently suggested that defective activation of STAT1 in fibroblasts from psoriatic patients could be related to the physiopathology of the disease.

Taken together, our study is consistent with a role for fibroblasts in the regulation of skin inflammation (10) and the hypothesis that chronic inflammation may persist as a result of failure of the processes involved in tissue repair (19). Considering that fibroblasts maintain their topographic differentiation in culture (5), our results suggest that psoriatic fibroblasts could undermine the ability to correctly solve any tissue damage occurred in periods of remission. Therefore, fibroblasts represent a very interesting target in the treatment of chronic inflammatory diseases, such as psoriasis. For this, it is essential to deepen in the characterization of psoriatic fibroblasts from lesional and uninvolved skin, looking for potential epigenetic mechanisms responsible for changes in stromal cell phenotype and their significance in disease initiation and progression.

## Data Availability

The datasets generated for this study are available on request to the corresponding author.

## Author Contributions

JA performed the research and drafted the manuscript. RMA contributed to the design, analysis and interpretation of PCR experiments. AM-C contributed to the analysis and interpretation of macrophages experiments. MP made substantial contributions to the study design and data analysis. FV-C contributed with essential samples and critically revised the manuscript for important intellectual content. MCT and MCM designed the study, were major contributors to data analysis and interpretation and wrote the paper.

### Conflict of Interest Statement

The authors declare that the research was conducted in the absence of any commercial or financial relationships that could be construed as a potential conflict of interest.
