# Supplementary material for: Defective Induction of COX-2 Expression by Psoriatic Fibroblasts Promotes Pro-inflammatory Activation of Macrophages
Source: Front Immunol. 2019 Mar 20;10:536. doi: 10.3389/fimmu.2019.00536 (PMC6448046; doi:10.3389/fimmu.2019.00536)
Supplement: Supplementary file 1 [file Table_1.DOCX]

**Table S1. Demographic and clinical data of psoriatic patients**

| Patient | Sex | Age | BMI | PASI |
| --- | --- | --- | --- | --- |
| P1 | f | 22 | 23.5 | 3.6 |
| P2 | m | 40 | 30.5 | 4.2 |
| P3 | m | 43 | 33.9 | 10.8 |
| P4 | f | 56 | 23.7 | 9.6 |
| P5 | m | 33 | 21.5 | 6.0 |
| P6 | f | 48 | 24.8 | 1.4 |
| P7 | m | 60 | 31.6 | 4.8 |
| P8 | f | 39 | 30.8 | 2.4 |
| P9 | m | 27 | 29.5 | 26.6 |
| P10 | m | 40 | 27.4 | 3.0 |
| P11 | m | 59 | 27.2 | 6.2 |
| P12 | m | 20 | 17.7 | 2.4 |
| P13 | m | 76 | 30.0 | 60.6 |
| P14 | m | 37 | 50.3 | 1.8 |
| Mean ± SD |  | 42.9±15.7 | 28.7 ± 7.6 | 10.2 ± 15.9 |

**BMI:** body mass index; **PASI:** psoriasis area severity index
